# Work and diet-related risk factors of cardiovascular diseases: comparison of two occupational groups

**DOI:** 10.1186/1745-6673-5-4

**Published:** 2010-03-22

**Authors:** Danielle Hartung, Martina Stadeler, Romano Grieshaber, Sylvia Keller, Gerhard Jahreis

**Affiliations:** 1 Research Centre of Applied System Safety and Occupational Medicine, Erfurt, Mannheim, Germany; 2 Berufsgenossenschaft Nahrungsmittel und Gaststätten, Prevention Department, Mannheim, Germany; 3 Institute of Nutrition, University of Jena, Germany

## Abstract

**Background:**

Although work related risk factors associated with Cardiovascular Diseases (CD) have been well researched, there is no detailed knowledge regarding disparate occupational groups each with a different risk exposition. Therefore, two occupational groups (chefs and office workers) were compared with a focus on nutritional and psychosocial factors.

**Methods:**

Two groups of subjects were tested for work and diet-related risks of CD (45 chefs and 48 office workers). The groups matched both for gender (male) and age (30 to 45 years). The study included a medical check-up, bioelectrical impedance analysis as well as an evaluation of questionnaires on health, nutritional behaviour and coping capacity. In addition, volunteers were required to compile a 7-day-dietary-record and collect their urine 24 h prior to their check-up. Blood samples drawn were analysed for glucose and lipid metabolism, homocysteine, vitamin B_12_, folic acid; C-reactive protein, uric acid, red blood cell fatty acids, plant sterols, antioxidative capacity and oxidative stress.

**Results:**

On average, the chefs showed one risk factor more compared to the office workers. The most frequent risk factors in both groups included overweight/obesity (chef group [CG]: 62.2%; office group [OG]: 58.3%) and elevated TC (CG: 62.2%; OG: 43.8%]. Moreover, although the chefs often had higher CRP-concentrations (40.0%), more office workers suffered from hypertension (37.5%).

Chefs showed significant higher concentrations of saturated fatty acids and oleic acid, whereas docosahexaenoic acid, Omega-6- and *trans *fatty acids were found more frequently in the red blood cell membranes of office workers. While there were no significant differences in analysed plant sterols between the two occupational groups, 7,8-dihydro-8-oxo-2'-deoxyguanosine was significantly increased in office workers.

Concerning the work-related psychosocial factors, the chefs were characterised by a stronger subjective importance of work, a greater degree of professional aspiration and enhanced efforts at perfectionism at their workplace.

**Conclusions:**

The chefs in the study bear a higher risk of CD compared to the office-workers. Although, CD is not exclusively a result of workplace-conditions, study results show that work-related influences can not be ignored. Thus, prevention of CD may be an important task attributable to occupational physicians.

## Background

Atherosclerosis due to inappropriate nourishment together with a lack of physical activity is responsible for of approximately half of all the deaths of adults aged over 60 in industrialized nations worldwide [1].

Compared to former generations, today there is a readily available greater food supply and less physical activity in leisure and labour time. However, people today are confronted more frequently with complex psychosocial demands [2].

In addition to genetic and lifestyle factors, work-related influences are linked to a higher risk for diseases. Disability-statistics available from German Health Insurance Funds provide an insight into the prevalence of work-related risks of CD, yet such data gives no evidence of disease pathogenesis.

Chefs were chosen as a subject group for this study in the interest of the Preventive Department of the German Employers' Liability Insurance Association for Food Industry and Restaurants (Berufsgenossenschaft Nahrungsmittel und Gaststätten). The aim was to investigate whether chefs have a higher cardiovascular risk due to their job requirements. Certain aspects of their occupation such as the irregular working hours, erratic mealtimes and consumption of rich food may lead to a higher disease risk due to a higher energy intake, in particular of fat. Furthermore, psychosocial factors leading to mental stress instigated by working under time pressure during peak periods and complaints from discontented customers may additionally add to the risk of disease.

Regarding the work-related exposition of chefs, the focus was placed on atherosclerotic risk factors affected by nutrition. In addition to the analysis of the common risk parameters such as abnormal fasting glucose value and lipid metabolism, there are a large number of blood tests which can demonstrate the CD risk. Detailed parameters of lipid metabolism, especially lipoprotein (a) (Lp(a)) and apolipoprotein B (Apo B) were of interest to the study. The thrombogenic and atherogenic effects of Lp(a) are due to its close homology with plasminogen [3], whereas Apo B is the primary apolipoprotein of LDL responsible for carrying cholesterol to tissues and can be used to estimate the particle number and size of LDL. Since a high value of Apo B is related to heart disease, Apo B allows a more accurate assessment of CD risk [4].

High concentrations of the amino acid homocysteine have been implicated in the progression of CD owing to its association with a large number of atherogenic effects such as degenerated vascular architecture and endothelial function, elevated oxidative stress, and higher risk of thrombosis. Homocysteine, resulting as an intermediate product of the methionine metabolism, is re-methylated in presence of folic acid and cobalamin (vitamin B_12_). Therefore, an intake of both vitamins is necessary to prevent the accumulation of higher levels of homocysteine [5] since in addition to age, gender and rare genetic disorders, elevated levels of homocysteine are associated with a deficit of folic acid and cobalamin (vitamin B_12_) [6].

Uric acid is also discussed as a risk factor as it increases the blood pressure due to its stimulating effect on the proliferation of smooth vascular muscle cells and the activation of circulating platelets [7].

Finally, inflammatory processes are believed to play a role in the development of atherosclerosis. The measurement of C-reactive protein, a highly sensitive marker of inflammation provides a quick and a simple method of risk prediction [8].

Other risk factors associated with nutrition, include the fatty acids that are a part of the lipids regulating the structure and function of biological membranes [9]. Therefore, they can function as adequate biomarkers to monitor for the type and number of fatty acids ingested during the last 60-80 days [10,11].

For a long time saturated fatty acids (SFA) have been categorised as atherogenic because of increasing LDL-C levels although current case-control-studies give no significant evidence for this consideration. In fact, SFAs even seem to have a positive influence on HDL-C, when they are exchanged with carbohydrates on an isocaloric basis [12,13].

Omega-3 fatty acids are the most important fatty acids having a preventive function for CD. Both eicosapentaenoic (EPA) and docosahexaenoic acid (DHA) are e.g. found in fish oil or can be synthesised from the essential alpha linoleic acid (ALA). The synthesis of the two Omega-3 fatty acids is dependent on the nutritive supply of ALA, found in vegetable oil, e.g. in linseed, hempseed and walnut oil [14]. The protective effects of Omega-3 fatty acids include a positive influence on lipoprotein metabolism, blood pressure and glucose tolerance as well as the anti-inflammatory and anti-thrombotic effects [15].

In contrary *trans *fatty acids (TFAs) are associated with a higher risk of CD due to hazardous effects such as negative influences on lipoprotein metabolism, pro-inflammatory effects, elevation of oxidative stress as well as deteriorated fluidity of membranes and insulin sensitivity et cetera[16].

Sterols are essential components of cellular membranes of plants differing from cholesterol by possessing an additional methyl or ethyl group. They have positive influence on human cholesterol levels as they compete for the same resorption protein (Nieman-Pick C1 like 1 protein) and are selectively removed back to the intestine [17].

Both oxidative stress and the concentrations of antioxidants provide additional information concerning the risk of CD. Oxidative stress contributes to atherogenesis due to oxidation of LDL-C, thereby influencing the genesis of many degenerative diseases [18]. The human body possesses an effective defence against reactive oxygen species which can additionally be supported by an exogenous supply of antioxidants. However, the positive effect of exogenous antioxidants on oxidative stress is controversial and in fact, current studies indicate negative effects due to an excessive supply of antioxidants [19]. A means of assessing oxidative stress is offered by measuring levels of the biomarker 7,8-dihydro-8-oxo-2'-deoxyguanosine (8-oxodG) in urine. Antioxidant levels can be analysed using alpha-Tocopherol, total phenolics (measured as gallic acid equivalents (GAE)) as well as equivalent antioxidant capacity III (TEAC III).

## Subjects and Methods

### Subjects

The study groups consisted of chefs (N = 45) and office workers (N = 48). All subjects were male and aged between 30 and 45 years. The volunteers were informed of the purpose, course and possible risks of the study and all subjects signed the consent form. The study was approved by the ethics committee of the Friedrich Schiller University (Jena, Germany).

### Procedure

Data from the chef group were collected between April 2004 and April 2005. The data form the group of office workers were collected between August and November 2005. All subjects underwent a detailed medical check-up with a bioelectrical impedance analysis. The volunteers were interviewed with regards to their common health state and nutritional pattern, including filling-out a food-frequency-questionnaire. By using the measure of coping capacity questionnaire (MECCA), items of work-related behaviour and work experience were determined. The subjects were required to compile a 7-day-dietary-record and to collect their urine 24 h in advance to the medical check-up.

### Blood and urine sampling

Blood samples were drawn between 7:00 and 8:00 a.m. by venipuncture into 9 monovettes (Sarstedt, Nümbrecht, Germany) after overnight fasting. Four of the monovettes contained EDTA as an anticoagulant for plasma preparation. Blood samples were prepared and stored at -25° or -80°C until analyses.

Three urine-monovettes (Sarstedt, Nümbrecht, Germany) of 24-h urine were collected and stored at -25°C until analyses.

### Measurements

#### Clinical blood parameters

The following blood parameters were analysed in a clinical laboratory by using standardised methods: fasting glucose and HbA1c; total cholesterol (TC), high-density lipoprotein (HDL-C), low-density lipoprotein (LDL-C), triacylglycerides (TAG), Lp (a), ApoB; homocysteine, vitamin B_12_, folic acid; C-reactive protein (CRP) and uric acid.

#### Red blood cell (RBC) fatty acids and plant sterols

The analysis of red blood cell fatty acids was carried out qualitatively as follows: after initially isolating red blood cell membranes (RBCM) in phosphate buffer [20], the samples were washed and the lipids were educed from membranes by means of the BLIGH & DYER procedure [21] with methanol and chloroform. In preparation of the gas chromatographic (GC) analysis, the lipids were methylated for 60 min. at 80°C with methanolic hydrochloric acid (5% w/v). The resulting levels of fatty acid methyl ester (FAME) were analysed via GC after performing thin layer chromatography with hexane/diethyl ether/glacial acetic acid (85: 15: 0,2) [22].

The preparation and measurement of plant sterols in human plasma is described elsewhere [23].

#### Antioxidants and Oxidative stress

The measurement of hydropholic Trolox equivalent antioxidative capacity III (TEAC III) was carried out as described by Re et al[24]. Total phenolics were analyzed spectrophotometrically (750 nm) by using a modified Folin-Ciocalteu method [25]. The sample preparation was performed according to Serafini et al. [26]. The measurement of plasma α-tocopherol, retinol and urinary 7,8-dihydro-8-oxo-2'-deoxyguanosine is described elsewhere [27].

#### Measure of coping capacity questionnaire (MECCA)

In order to determine the styles of coping with occupational burden, the measure of coping capacity questionnaire (MECCA) [28] was employed. It is a means of collecting information regarding behaviour and work experience that may be beneficial or hazardous to workers health. The items in the questionnaire refer to work-related dedication and endurance compared to burden and emotions at work. These items are categorised by a five-step rating scale and can be differentiated via cluster analysis into four patterns: a healthy-ambitious coping style (type G; German: Gesundheit = health), an unmotivated coping style (type S; German: Schonung = easy going), a pattern suffering from burnout (type B) and a pattern suffering severe strain (type A; German: Arbeit = work). Type A, associated with higher risk for CD, is characterised by excessive work-related dedication and the lowest ability to put work at a distance, with concomitant low peace of mind and mental equilibrium. In addition, type A is often associated with negative emotions which, in turn, have pathogenic consequences [29].

### Statistical methods

Statistical analysis were performed using SPSS for Windows, Version 11.5.1 (November 2002, SPSS Inc., Chicago, USA). The assessment of normal distribution was carried out by Kolmogorov-Smirnov test. P < 0.05 was considered as significant. Due to not normally distributed variables, values are presented as the median and the 25^th ^or the 75^th ^percentile (P25-P75), respectively. The comparison of the professional groups was analysed using Mann-Whitney-U test. Pearson's χ^2 ^test was used to estimate the frequency of distribution in subgroups. Fisher's exact test was applied in subcategories with n < 5. Coefficient of rank correlation was calculated by Spearman.

## Results

### Anthropometric data and dietary intake, smoking habits

There were no significant differences in BMI, body fat and lean body mass (chefs 68.1 kg versus office worker 69.0 kg; see Table 1) between the two occupational groups. In essence, a similar outcome was also achieved from the dietary records. However, a significant higher intake of fibres (17.7 g versus 21.3 g) and tocopherol (9.0 g versus 11.3 g) was obtained from the records for the office group. The energy intake for both groups was within recommended values of average energy input (ca. 2650 kcal/d). Protein- (96.6 g/d versus 90.9 g/d) and liquid intake (2915 ml versus 2682 ml) in the chef group was slightly, but not significantly increased. Further, smoking habits in the chef group was twice as high compared to the group of office workers (Table 1).

**Table 1:** Prevalence and medians of CD-risk factors of the compared occupational groups

Prevalence of elevated risks					median (P25-P75)		p^1^
Group	chef group		office group		chef group	office group	
	N	%	N	%	N = 45	N = 48	
Physical and mental health							
BMI > 25 kg/m^2^	28	62.2	28	58.3	26.1 (22.8-28.1)	25.3 (22.6-27.4)	NS
body fat > 22%	16	35.6	16	33.3	19.0 (15.0-24.0)	19.5 (15.0-22.8)	NS
blood pressure > 130:85 mmHg	16	35.6	18	37.5			
MECCA type A^3^	15	33.3	5	10.4			0.001^2^
							
lifestyle factor: smoking							
smokers	16	35.6	8	16.7			0,037^2^
							
blood parameters							
fasting glucose > 6.4 mmol/l	5	11.1	1	2.1	5.2 (4.7-5.7)	5.4 (5.1-5.6)	NS
HbA1c > 6.1%	1	2.2	0	-	5.3 (5.0-5.6)	5.2 (5.0-5.4)	NS
TC > 5.2 mmol/l	28	62.2	21	43.8	5.5 (4.9-5.9)	5.1 (4.3-5.7)	0.045
LDL-C > 3.9 mmol/l	15	33.3	11	22.9	3.5 (3.0-4.3)	3.5 (2.8-3.9)	NS
HDL-C < 0.9 mmol/l	6	13.3	6	12.5	1.3 (1.1-1.5)	1.2 (1.0-1.3)	NS
TAG > 1.7 mmol/l	12	26.7	13	27.1	1.2 (0.8-2.0)	1.0 (0.7-1.8)	NS
Lp (a) > 30 mg/dl	11	24.4	10	20.8	7.9 (4.8-32.2)	7.3 (2.4-25.6)	NS
ApoB > 1.6 g/l	0	-	0	-	1.1 (1.0-1.3)	0.9 (0.7-1.1)	0.002
CRP > 3 mg/l	18	40.0	11	22.9	2.0 (1.0-4.5)	2.3 (1.0-3.0)	NS
uric acid > 420 μmol/l	7	15.6	1	2.1	354 (308-389)	322 (302-363)	0.018
homocysteine > 9.2 mmol/l	8	17.8	12	25.0	8.0 (6.0-9.0)	8.0 (6.8-9.3)	NS
vitamin B_12 _< 165 pmol/l	1	2.2	1	2.1	265 (228-335)	245 (203-285)	0.027
folic acid < 3.6 ng/ml	2	4.4	4	8.3	7.7 (5.8-10.0)	6.5 (5.4-8.7)	NS

^1 ^Mann-Whitney-U test

^2 ^Pearson's χ^2 ^test

^3 ^for details see text

### Clinical blood parameters and risk stratification

When all relevant risk factors (factors with defined references or evidenced relation to CD) were accumulated, a significant difference between the occupational groups was revealed (Mann-Whitney-U test, p = 0.029). On average, chefs showed one more risk factor than office workers (5 versus 4). The most frequent risk factors in both groups were overweight/obesity and increased TC. Furthermore, chefs often had higher CRP-concentrations whereas more office worker suffered from hypertension (Table 1).

Chefs had significant higher concentrations of uric acid. They also featured better vitamin B_12 _and folic acid-states and therefore, showed fewer cases of increased homocysteine-concentrations (Table 1).

### Red blood cell fatty acids and plant sterols

Approximately 50% of analysed FAMEs were saturated. Chefs showed significant higher concentrations of SFAs in the membranes of red blood cells in comparison to office workers. Concerning Omega-3-fatty acids, significant differences were only found between the two occupational groups regarding DHA. Office workers had higher ratios at the most Omega-6-fatty acids too (linoleic acid [LA], arachidonic acid [AA]). In contrast, oleic acid (OA), the most prevalent Omega-9-fatty acid occurred more significantly in the red blood cells of chefs (Table 2). There were no significant differences in the plant sterol concentrations between the monitored groups (Table 2).

**Table 2:** Medians and percentiles of descriptive CD-parameters of the compared occupational groups

	chef group	office group	p^1^
RBC fatty acids	N = 45	N = 48	
SFA (% of FAME)	50.43 (47.37-53.19)	47.93 (45.71-51.15)	0.019
ALA (% of FAME) [Omega-3-FA]	0.11 (0.08-0.21)	0.14 (0.12-0.16)	NS
EPA (% of FAME) [Omega-3-FA]	0.29 (0.15-0.53)	0.37 (0.19-0.56)	NS
DHA (% of FAME) [Omega-3-FA]	1.05 (0.53-2.17)	2.29 (1.72-3.00)	0.000
LA (% of FAME) [Omega-6-FA]	9.04 (8.06-10.10)	10.07 (9.62-10.89)	0.000
AA (% of FAME) [Omega-6-FA]	6.86 (4.79-9.57)	10.58 (6.72-12.34)	0.000
OA (% of FAME) [Omega-9-FA]	16.89 (15.80-18.50)	16.04 (15.23-17.26)	0.013
Σ TFA (% of FAME)	0.25 (0.19-0.36)	0.37 (0.28-0.48)	0.000
			
Plant sterols	N = 44	N = 47	
Campesterol (μg/ml)	3.04 (2.59-3.74)	3.41 (2.93-5.42)	NS
Sitosterol (μg/ml)	2.99 (2.73-3.51)	2.72 (2.32-3.71)	NS
			
Antioxidants	N = 45	N = 48	
TEAC III - hydrophilic (mmol/l)	2.61 (2.47-2.82)	2.88 (2.77-3.01)	0.000
Total phenols measured in GAE (mg/l)	1151 (1040-1253)	947 (880-998)	0.000
α-Tocopherol (μmol/l)	22.22 (20.28-27.38)	18.40 (15.82-21.77)	0.000
			
Oxidative stress	N = 43	N = 46	
8-oxodG nmol/mmol creatinine)	0.52 (0.40-0.74)	0.72 (0.48-1.00)	0.017

^1 ^Mann-Whitney-U test

### Antioxidants and oxidative stress

Chefs showed significant lower hydrophilic Trolox equivalent antioxidative capacity (TEAC III) in plasma than office workers, but in contrast significant higher total phenolic (GAE) levels. Significant higher concentrations of plasma α-tocopherol were detected in the chef group (Table 2). The biomarker for oxidative stress, 8-OxodG was present at a significant higher rate [nmol/mmol creatinine] in the 24-h-collected urine of the office group (Table 2).

### Risk caused by mental stress

A remarkable difference between the occupational groups was found on evaluating the MECCA questionnaire. Although 50% of chefs showed the healthy-ambitious coping style type G, they were significantly more often assigned to the CD-related type A than office workers (Pearson's χ^2 ^test; Table 1). Chefs differed in all dimensions of work-related dedication: they featured a greater willingness for strenuous work, more occupational ambition and efforts at achieving perfection. Work was more important for the chefs and they were less capable of mentally distancing themselves from their work than the office workers.

## Discussion

### Risk and nourishment

It was hypothesised that due to their working conditions, chefs endorse a nutritional state and behaviour that is not beneficial for cardiovascular health. At first sight, however, there was no evidence to support this hypothesis as body weight, body fat, and lean body mass, were not different to the comparison group. Moreover, the findings from the 7-day-dietary-records in the two groups showed no specific differences, except for the significantly lower fibre and vitamin-E intake in the chef group. However, a detailed analysis revealed contrary findings, which could be attributed to the differences in nutrition and lifestyle between the investigated groups.

In the chef group, significantly increased concentrations of parameters associated with higher intake of meat and animal fats and a lower intake of vegetable and fruits as well as whole-grain products were found. These include TC, Apo B, vitamin B_12 _and uric acid together with a higher fraction of SFA in RBC. The elevated concentrations of Apo B indicate a higher levels of small, dense LDL-C, which is known to aggravate atherosclerosis [4,30]. The lower intake of fibres and a slightly higher intake of proteins support the assertion that chefs consume more animal products. This statement is also confirmed by the food-frequency-questionnaire in which subjects in the chef group admitted eating more meat (Fisher's exact test, p = 0.002) and less vegetables and fruits than office workers. Due to this diet, chefs had a better supply of vitamin B_12 _and therefore, a lower risk of increased homocysteine concentrations.

### Socio-economic factors

Diet and intake of nutrient are influenced by the social and economic condition of the populace. Studies have revealed that individuals with a lower level of education and/or income consume less fish and vegetables, but more fried foods, pasta, potatoes, table sugar and beer. Hence, they ingest fewer vitamins and minerals (e. g. vitamin A, iron and calcium) while their intake of fat, SFAs as well as carbohydrates, especially simple sugars are considerably higher. All these facts indicate an inferior nutritional behaviour with all its negative effects on the risk of CD such as metabolic syndrome etc [31-34].

The higher percentage of DHA in the RBCM of office workers might be explained by the higher socio-economic situation in this group. Dietary supplements can be ruled out as being causal since no subject supplemented with Omega-3-fatty acids.

Similar results were found in the *Heart and Soul Study*. Patients with coronary artery diseases and a lower socio-economic status defined according to household income, education, occupation, and housing status showed lower concentrations of DHA and EPA in RBCM. The authors state that this was due to a lower fish consumption [35]. However, according to the answers given in the food frequency questionnaire in this study, the chefs consumed more fish in comparison to the office workers (Fisher's exact test, p = 0.002). Though the nature of this profession allows chefs to consume more fish, it is moot whether they actually eat more fish. The preference for meat in this sample of chefs seemed to be more important. Therefore from this finding are no consequences regarding CD-risk derivable.

### Fatty acids

The Omega-6-fatty acids in RBCM were also significantly different between the two observed occupational groups. A higher percentage of LA as well as AA were revealed in RBCM of office workers. Increased levels are caused by a higher intake of fatty acids in food or by the conversion of LA to AA. In a normal Western diet, the conversion too AA usually exceeds the dietary supply of AA [36]. However, the office workers could have consumed more LA for example in the form of LA-rich oils or by the eating of nuts that are responsible for higher percentages of Omega-6-fatty acids in RBCM [37].

A higher intake of TFA-rich food (e. g. chips, instant products such as soups and sauces) and therefore, an increased concentration of TFA were expected in the RBCM from the chefs. On the contrary, the chefs showed a significant lower sum of TFA compared to the office group. Thus, there is no higher risk resulting from TFAs for chefs.

### Plant sterols

The same levels of plant sterol concentrations found in the two groups might be explained by a lesser influence of nutrition and lifestyle on plant sterol concentrations in plasma. Due to the fact that the intake of plant sterols in a common Western diet, estimated to be between 200 - 400 mg/d, is potentially to low for the accumulation of plant sterols in plasma, assumed that individuals are not suffering from genetic disorders like phytosterolemia [17,38].

However, is there at all a need to enrich food with plant sterols? Current research in this field provides controversial data. For example, individuals with phytosterolemia have a higher risk of CD because of the atherogenic potential of plant sterols in higher plasma concentrations [39,40]. It is common that subjects with phytosterolemia also suffer from hypercholesterolemia and may be preferentially consuming food products enriched with plant sterols, under the assumption that they are improving their lipid profile. However, due to a lack of study data, it is difficult to estimate the prevalence of phytosterolemia in the population. Thus, there is a need of more, and in particular longer term studies [41].

### Oxidative stress

The results correlating to the analysis of oxidative stress and the concentrations of antioxidants in plasma were unexpected. Office workers either smoked less or only occasionally. Moreover, they consumed more vegetables and fruits in comparison to chefs. Nevertheless, this group showed significantly higher concentrations of 8-oxodG in the 24-h-urine samples. The Spearman correlation did not show an association between 8-oxodG and the hydrophilic TEAC III in plasma (data not shown). Thus, antioxidative capacity in food does not correlate very well with those of individuals and may be influenced by several as yet not well understood factors such as bioavailability, absorption, metabolism and the antioxidative capacity of different total phenols in vivo. Further studies are needed to determine the effect of antioxidants [42].

The inverse relationship of TEAC III and total phenols between the two occupational groups can be explained by an increased antioxidative capacity without a concomitant increase in total phenol levels. The last one may depend on varying composition of polyphenols in plasma [43]. Furthermore phenols in plasma can be influenced by the amount of other antioxidants (e. g. vitamin C) in plasma [44]. On the other hand, a diet rich in polyphenols does not necessarily enhance GAE-concentrations, but can increase the antioxidative capacity [45].

### Psychological factors

The remarkably high score of MECCA type A in the chef group was comparable to the teaching profession, which has scored the highest percentage of MECCA risk types A and B (30% in each case) of all occupational groups analysed to date [29].

The results indicate that chefs encountered workplace-related stressors. This is confirmed by the results from the stress self-assessment test. The stress suffered by the chefs was more frequently job-related rather than resulting from other causes. They apparently responded to this stress-related situation by increasing their efforts at work which in turn, lead to a lesser ability to distance themselves from their work. The fear of job loss in the group of chefs seemed to be another reason for their tending to MECCA type A.

It is worth noting that a meta-analysis of fourteen prospective cohort studies demonstrated an average 50% excess risk for coronary heart diseases among employees with workplace stress [2,46].

## Conclusions

The chefs involved in the study carry a higher risk of CD than the comparison group of office workers. The nutritional behaviour of chefs consisting of food rich in fats, particularly animal origin is associated with higher TC, Apo B and higher rates of SFAs in red blood cell membranes as well as higher concentrations of uric acid. In addition, there were more smokers in the chef group.

Chefs have a stronger work-related dedication, which is displayed by a significant higher effort at achieving perfection, more willingness to perform strenuous work and an increased occupational aspiration. These factors can lead to psychological stress, which is strongly associated with a high risk of CD.

The differences which where found between the two groups are probably not only work-related. The study design and method were not able to appraise a clear relationship between workplace-conditions and analysed risk parameters. However, according to statements of the investigated chefs, their nutritional behaviour is not only work-related influenced but also chosen willingly.

Although CD are not only caused by workplace-conditions, work-related influences can not be ignored. The cooperation with Employers' Liability Insurance Associations and Health Insurance Companies as well as employers and employees is the base for a multi-causal approach for the prevention of CD. Occupational physicians provide the necessary link between the involved parties and are in an excellent position to give advice to both employers and employees regarding better working conditions, a healthier lifestyle (balanced diet, stop smoking, physical activity, stress coping etc.) and provide an effective job motivation.

Finally, research on other occupational groups with reference to the risk of CD is necessary. A subsequent follow-up study after ten years is also essential to provide data on the progression of the risk in the reviewed groups.

## Abbreviations

(ALA): alpha linoleic acid; (AA): arachidonic acid; (CD): cardiovascular diseases; (DHA): docosahexaenoic acid; (EPA): eicosapentaenoic acid; (FAME): fatty acid methyl ester; (GAE): gallic acid equivalents; (LA): linoleic acid; (OA): oleic acid; (RBCM): red blood cell membranes; (SFA): saturated fatty acids; (TFA): trans fatty acids; (TEAC III): trolox equivalent antioxidant capacity III; (8-oxodG): 7,8-dihydro-8-oxo-2'-deoxyguanosine.

## Competing interests

The authors declare that they have no competing interests.

## Authors' contributions

DH and MS carried out the study, participated in the sequence alignment and drafted the manuscript. RG participated in the design of the study. SK participated as a counsellor relating to laboratory techniques and scientific background. GJ contributed to the study design and coordination and helped to draft the manuscript. All authors read and approved the final manuscript.
